# Epidemiology of ticks submitted from human hosts in Alberta, Canada (2000–2019)

**DOI:** 10.1080/22221751.2022.2027217

**Published:** 2022-02-01

**Authors:** Jamil N. Kanji, Abraam Isaac, Daniel Gregson, Monika Mierzejewski, Danny Shpeley, Pauline Tomlin, Michael Groeschel, L. Robbin Lindsay, Lisa Lachance, Kinga Kowalewska-Grochowska

**Affiliations:** a Division of Infectious Diseases, Department of Medicine, University of Alberta, Edmonton, Canada; b Department of Laboratory Medicine and Pathology, Faculty of Medicine and Dentistry, University of Alberta, Edmonton, Canada; c Public Health Laboratory, Alberta Precision Laboratories, University of Alberta Hospital, Edmonton, Canada; d Diagnostic and Scientific Centre, Alberta Precision Laboratories, Calgary, Canada; e Department of Pathology & Laboratory Medicine, Cumming School of Medicine, University of Calgary, Calgary, Canada; f Division of Infectious Diseases, Department of Medicine, Cumming School of Medicine, University of Calgary, Calgary, Canada; g Department of Biological Sciences, University of Alberta, Edmonton, Canada; h One Health, National Microbiology Laboratory, Public Health Agency of Canada, Winnipeg, Canada; i Department of Microbiology, Immunology, and Infectious Diseases, Cumming School of Medicine, University of Calgary, Calgary, Canada; j Alberta Health, Public Health and Compliance Division, Health and Wellness Promotion Branch, Edmonton, Canada; k Department of Medical Microbiology & Immunology, Faculty of Medicine, and Dentistry, University of Alberta, Edmonton, Canada

**Keywords:** Tick, ixodes, dermacentor, lyme, Borrelia burgdorferi, alberta

## Abstract

The geographic range and occurrence of tick species is dynamic. This has important public health implications due to important tick species that can transmit pathogens. This study presents a retrospective review of tick genera recovered from humans and submitted for identification in Alberta, Canada, over a 19-year period. The total number of ticks and proportion of genera were analyzed over time. Molecular testing for a number of pathogens associated with *Ixodes scapularis* and *I. pacificus* was conducted. A total of 2,358 ticks were submitted between 2000 and 2019, with 98.6% being acquired in Alberta. The number of ticks submitted increased significantly over time (*p *< 0.0001). *Dermacentor* ticks were the most abundant genus, followed by *Ixodes* and *Amblyomma*. There was a significant decrease in the proportion of *Dermacentor* ticks between 2013 and 2019 (*p *= 0.02), with a corresponding increase in the proportion of *Ixodes* ticks over the same time (*p *= 0.04). No statistically significant change in seasonality was identified. *Borrelia burgdorferi* was detected in 8/76 (10.5%; 95% CI 5.4–19.4%) of all *I. scapularis* and *I. pacificus* ticks submitted. This translated to a *B. burgdorferi* positivity of 0.35% (95% CI 0.15–0.68%) among all ticks received. *Dermacentor* species (especially *D. andersoni*) remains the most common tick feeding on humans in Alberta. Small numbers of vector species (including *I. scapularis/pacificus*) are encountered annually over widely separated geographic areas in the province. The risk of exposure to tick-borne pathogens (e.g. Lyme disease) in Alberta remains low.

## Introduction

Ticks constitute important vectors of several zoonotic pathogens. The distribution of ticks and pathogens they may transmit vary based on species, as well as geographic location [1]. *Borrelia burgdorferi*, *Anaplasma phagocytophilum, Babesia microti,* and Powassan virus are agents of tick-borne zoonoses transmitted by species of *Ixodes* in North America [2,3].

Expansion of the geographic distribution of tick species is multifactorial, including climate change, abundance in source locations, and capacity to be spread by the host (such as northwards expansion via bird migration) [2,4,5]. One study of ticks from migratory birds found that up to 22 tick species had been introduced into Canada, some originating as far south as Brazil [6]. Once on Canadian soil, these ticks have the potential to spread through the ecosystem; and, in some instances, introduce novel zoonotic pathogens into local tick populations [7,8].

Tick surveillance in the province of Alberta, Canada, up until 2021 has been conducted via two methods. The primary methodology is passive surveillance, where ticks are submitted by veterinarians and the public themselves. The second methodology is active tick surveillance, in which ticks are collected from the environment, typically by drag sampling, and the locations to actively sample are often triggered by signals from passive tick surveillance. Veterinarians and the public are encouraged to submit ticks found in the environment or on humans or companion animals for identification. All submitted species of *Ixodes* ticks (except *I. kingi* and *I. ochotonae*) are tested for selected zoonotic pathogens, including *B. burgdorferi*. Passive surveillance of ticks from veterinary and environmental sources from 2013 to 2019 revealed a mean annual submission of 1,465 ticks per year (no travel outside the province). Of these, 10–11% each year were found to be *I. scapularis* or *I. pacificus*, with an average of 14.4% found to be positive for *B. burgdorferi* [9].

Active tick surveillance was last conducted in Alberta annually from 2014 to 2017 at various sites throughout the province. During this time, no ticks were identified. No active surveillance was conducted in 2018 and 2019, given insignificant findings on passive surveillance during those years [9].

Outside of these surveillance programs, clinicians have also been able to submit ticks extracted from human patients to diagnostic microbiology laboratories in Alberta since 2000. All ticks were identified to the genus (and, if possible, species); providing important clinically relevant information necessary for counseling patients with anxiety post-tick-bite. This subset of data was not captured locally by established passive surveillance programs, thus providing valuable information to complete data for all human and animal hosts for the province.

To better understand the epidemiology of ticks acquired from human hosts in Alberta, we undertook a retrospective review of tick identifications conducted by diagnostic microbiology laboratories in Alberta over a 19-year period. Of particular interest to clinicians and Public Health was to estimate the likelihood that a tick removed from a human host in Alberta carries *B. burgdorferi*, as this is a very common query from patients and the public that arises while awaiting identification of the tick that is often difficult to address.

## Methods

### Population and study design

The province of Alberta in Western Canada has a population of approximately 4.5 million; [10] and for the purposes of healthcare delivery, is divided into five zones: North, Edmonton, Central, Calgary, and South Zones [11]. Since 2000, clinicians have been able to submit ticks brought in by patients to diagnostic microbiology laboratories for identification [12]. Each submission is accompanied by a requisition requesting detailed history, including relevant travel. Ticks found in the environment or from non-human hosts were not accepted by diagnostic microbiology laboratories and instead redirected to other surveillance avenues [9].

### Tick identification and pathogen detection

Each arthropod was identified using morphological identification keys [13]. All ticks identified as *Ixodes* spp. were then forwarded to the Provincial Public Health Laboratory for confirmation and, if necessary, for second confirmation by the Department of Biological Sciences at the University of Alberta.

All *Ixodes spp.* were subsequently sent to the National Microbiology Laboratory (Public Health Agency of Canada, Winnipeg, Manitoba) for pathogen testing. Specimens of *I. scapularis* and *I. pacificus* were tested for infection with *Borrelia burgdorferi*, *Borrelia miyamotoi*, *Anaplasma phagocytophilum*, and *Babesia microti* by real-time PCR as previously described [14]. Briefly, QIAGEN DNeasy 96 tissue kits (QIAGEN Inc., Mississauga, Ontario) were used for DNA extraction. A duplex screening assay was chosen to screen the samples for *Borrelia* spp. using the 23S rRNA real-time polymerase chain reaction (PCR) assay, and *Anaplasma phagocytophilum* using the msp2 real-time PCR assay [15]. Analysis for *Babesia microti* was conducted using the methods described by Nakajima et al. for the detection of the CCT eta gene [16]. All *Borrelia* spp.-positive samples were subsequently tested for *B. burgdorferi* using a confirmatory ospA real-time PCR assay, and *Borrelia miyamotoi* using an IGS real-time PCR assay. *Borrelia miyamotoi-*positive samples were further verified using the glpQ real-time PCR assay [14]. To account for possible contamination during extraction and the PCRs procedures, water or blank controls were included in all extractions and PCR, respectively.

### Data extraction and statistical analysis

A retrospective review of all ticks submitted for identification to diagnostic microbiology laboratories in Alberta from 1 January 2000 to 31 December 2019 was conducted. Information was extracted from multiple laboratory information systems used across the province and compiled. All retrievable scans or copies of original requisitions (dating back to 2000) were obtained from electronic archiving for review to ensure no travel history was missed during data entry. Records indicating travel from outside the province (national or international) were analysed separately; any specimen without a travel history was assumed to have been acquired from within Alberta.

Data was extracted in multiple formats and tabulated in Microsoft Excel. Proportional comparisons over time were conducted using the sum of squares linear regression modeling with statistical comparison of non-parametric variables using Chi-square analysis regression modeling (StatPlus, AnalystSoft Inc, Alexandria, USA). Significance was set at *p *< 0.05. Confidence intervals (CIs; 95%) were calculated using Wilson’s method.

## Results

### Numbers of ticks submitted

Between 1 January 2000 and 31 December 2019, a total of 2,358 ticks were submitted for identification, of which 32 had travel history from outside of the province (Supplementary Table S1). Based on this review, 2,326 ticks were deemed to be acquired within Alberta. The most common identified genera were *Dermacentor* (91.7%; 95% CI 90.5–92.3%), *Ixodes* (5.8%; 95% CI 4.9–6.8%), and *Amblyomma* (1.9%; 95% CI 1.5–2.6%). In total, 17.6% (95% CI 16.4–19.5%) of the ticks could only be identified to the genus level (Table 1).

**Table 1.** Identification of ticks deemed^a^ to be acquired within Alberta submitted from 2000 to 2018.

Genus (%)	Number (% of total ticks)
*Dermacentor*	2132 (91.7)
• *Dermacentor spp* – 338 (15.9)	
• *D. andersonii* – 1376 (64.5)	
• *D. variabilis* – 410 (19.2)	
• *D. albipictus* – 8 (0.4)	
*Ixodes*	134 (5.8)
• *Ixodes spp* – 43 (32.1)	
• *I. scapularis* – 67 (50)	
• *I. pacificus* – 11 (8.3)	
• *I. cookei* – 4 (3.0)	
• *I. angustus* – 1 (0.7)	
• *I. ricinus* – 4 (3.0)	
• *I. spinipalpis* – 1 (0.7)	
• *I. marxi* – 1 (0.7)	
• *I. muris* – 2 (1.5)	
*Amblyomma*	45 (1.9)
• *Amblyomma spp* – 22 (49.0)	
• *A. americanum* – 18 (40)	
• *A. maculatum* – 1 (2.2)	
• *A. cajennense* – 2 (4.4)	
• *A. coelebs* – 2 (4.4)	
*Boophilus spp.*	3 (0.1)
*Haemaphysalis leporispalustris*	1 (0.05)
*Ornithodros spp.*	1 (0.05)
*Rhipicephalus*	10 (0.4)
• *Rhipicephalus spp.* – 4 (40)	
• *R. sanguineus* – 5 (50)	
• *R. pulchellus* – 1 (10)	
Total	2326

^a^ Based on the detailed review of requisitions submitted with accompanying tick specimen. Abbreviations: spp. – notes species.

### Ticks submitted from within Alberta

**Figure 1.**
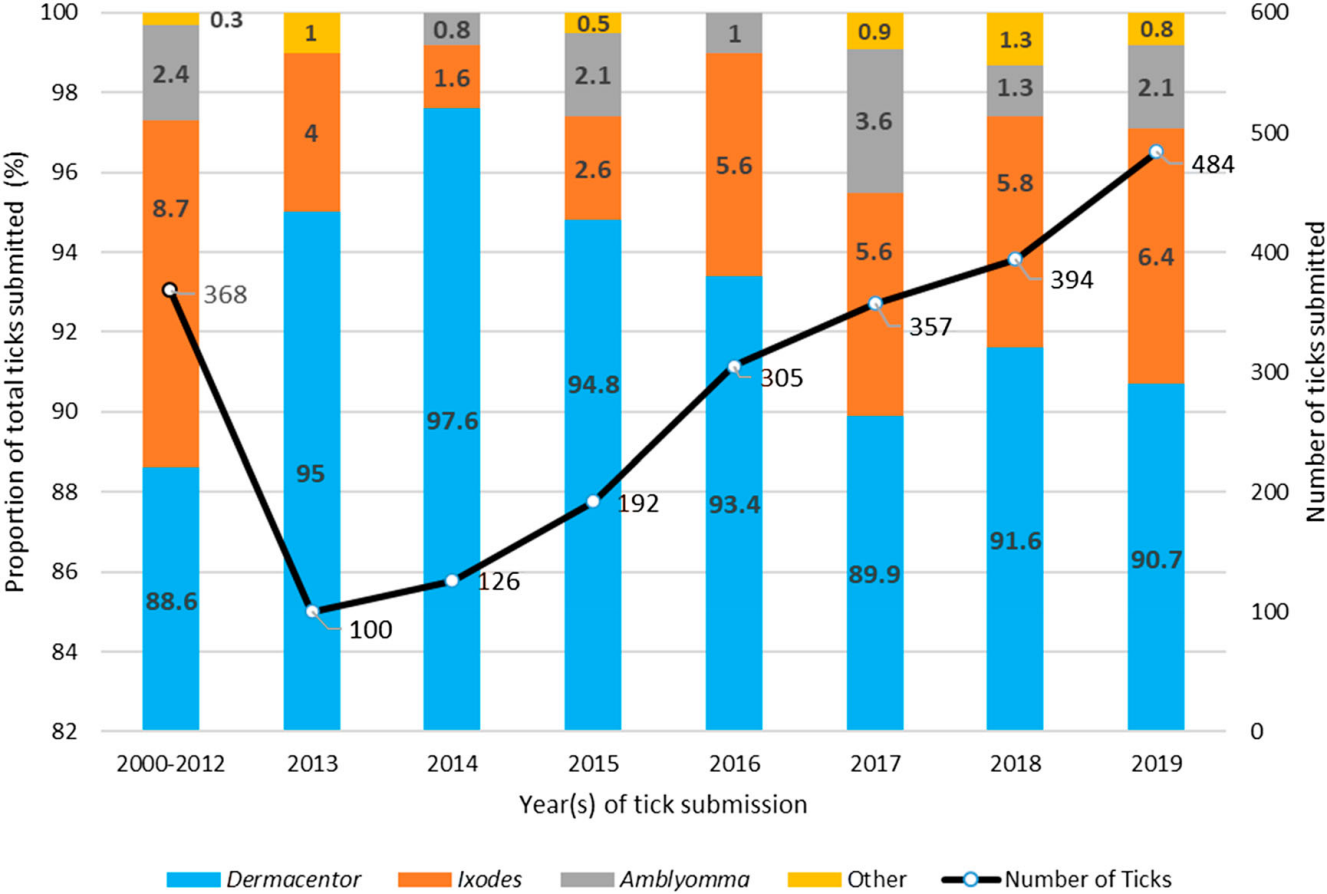
Number of ticks submitted over time and proportional representation of genera in teach time category (*n* = 2,326 ticks). Data values in bold font represent proportions in the specific year(s) indicated. Data values that are not bolded represent numbers of ticks submitted in the year(s) indicated.

In total, 2,326 (98.6%; 95% CI 98.1–99.0%) ticks were presumed to be acquired within Alberta. Due to the transition from paper to electronic laboratory records in 2013, ticks submitted from 2000 to 2012 have been grouped together as a cohort as a result of data extraction differences and availability. Between 2013 and 2019, the number of ticks has increased by four-fold (*p *< 0.0001) every year (Figure 1). The most common ticks identified annually were *Dermacentor* spp. followed by *Ixodes* spp. There was a significant increase in the proportion of *Ixodes* spp. submitted over the time period (*p *= 0.04) with a corresponding decrease in the proportion of *Dermacentor* spp. (*p *= 0.02), but no statistically significant change was noted for *Amblyomma* spp. (*p *= 0.18) and other genera (*p *= 0.42). Complete data regarding duplicate tick submissions or presence of immature forms (nymphs or larvae) was not consistently available.

### Geographic distribution of tick genera across the province

**Figure 2.**
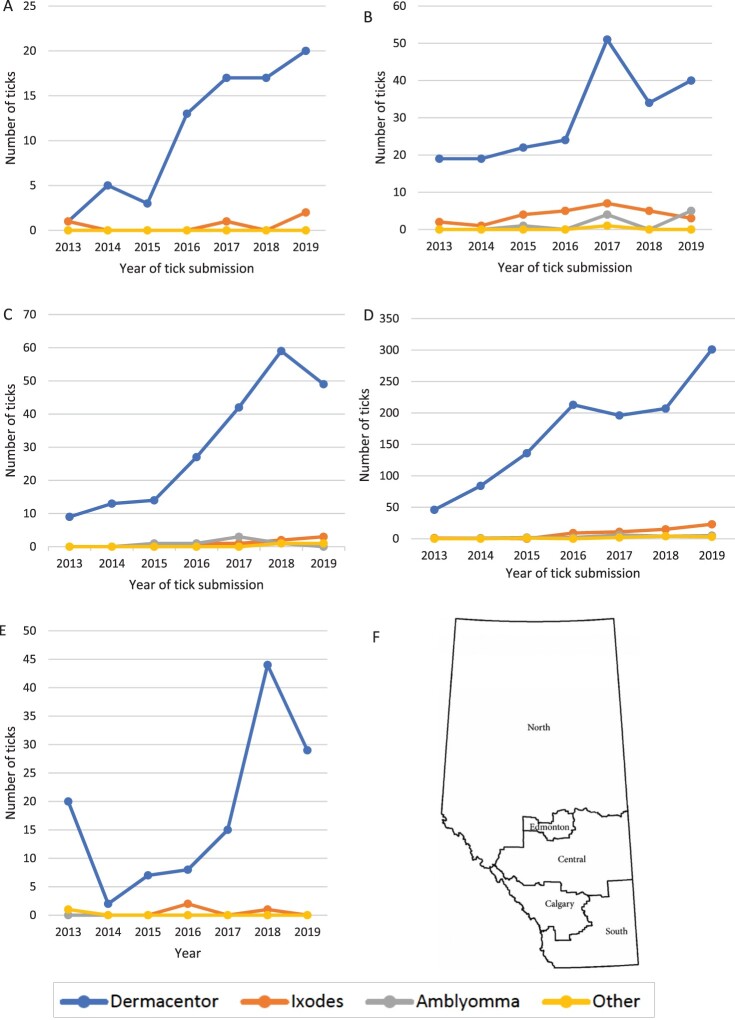
Numbers of each tick genera submitted from each Alberta health region based from 2013 to 2019: (a) North Zone; (b) Edmonton Zone; (c) Central Zone; (d) Calgary Zone; (e) South Zone; (f) Map of Alberta showing health zones. ^a^ Figure adapted from *Can Resp J* 2016; 1382434:1–9.

Data on Alberta Health zone of submissions was available for 1,958 (84.2%) of Alberta-acquired ticks from 2013 to 2019. A geographic representation of tick genera based on health zone unit designation is shown in Figure 2. *Dermacentor spp.* (*n* = 1806, 92.2%; 95% CI 91.0–93.3%) ticks were most common in all health zones annually. *Amblyomma* spp. (*n* = 36, 1.8%; 95% CI 1.3–2.5%) were only submitted from Edmonton, Central, and Calgary zones. Overall, 65.5% (95% CI 63.2–67.6%), 58.3% (95% CI 48.6–67.3%), and 55.5% (95% CI 39.6–70.5%) of ticks identified as *Dermacentor* spp.*, Ixodes* spp.*,* and *Amblyomma* spp. between 2013 and 2019 were from the Calgary Zone, representing approximately 31.1% of the population of Alberta (Supplementary Table S2) [10].

### Seasonality of tick submission

**Figure 3.**
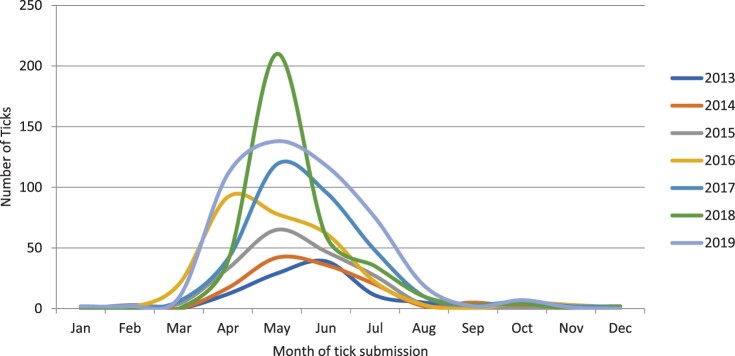
Seasonality of Alberta-acquired ticks submitted for identification from 2013 to 2018 (*n* = 1,876). Abbreviations: Jan – January; Feb – February; Mar – March; Apr – April; Jun – June; Jul – July; Aug – August; Sep – September; Oct – October; Nov – November; Dec – December.

The month of tick submission was available for 1,876 (80.7%) of Alberta-acquired ticks. Predictably, most tick submissions centered around the spring (March, April, and May) and summer (June, July, and August) seasons (Figure 3). Between 2016 and 2019, tick submissions increased by 7–10 ticks per fall season (September, October, November) per year. This, however, was not found to be statistically significant (*p *= 0.94). The overall increase in tick numbers noted in winter (*p *= 0.46), spring (*p *= 0.19), or summer (*p *= 0.20) was also not significant.

### Ixodes spp. ticks

Of the 2,326 ticks submitted from 2000 to 2019, 134 (5.8%; 95% CI 4.9–6.8%) were identified as *Ixodes* spp. Sixty-seven (50%; 95% CI 41.7–58.4%) were *I. scapularis,* eleven (8.2%; 95% CI 4.7–14.1%) *I. pacificus,* with the remainder identified as *Ixodes spp*. Molecular testing for *B. burgdorferi/Anaplasma phagocytophilum* DNA was conducted on 66/67 of the *I. scapularis* and 10/11 of the *I. pacificus* submissions. Of the ticks tested, 8/76 (10.5%; 95% CI 5.4–19.4%) (8/66 [12.1%; 95% CI 6.3–22.1%] *I. scapularis* and 0/10 *I. pacificus* ticks) were infected with *B. burgdorferi.* These ticks were identified between 2016 and 2019 (two in 2016; three in 2017; one in 2018; and two in 2019). Over this 4-year period, these positive *I. scapularis* ticks comprised 6.3–17.6% of the *Ixodes* spp. ticks submitted annually (Figure 2). Six of the *B. burgdorferi* positive ticks were from the Calgary zone and two from the Edmonton zone; the latter was also positive for *Anaplasma phagocytophilum*. *Anaplasma phagocytophilum* was also detected in three *B. burgdorferi*-negative *I. scapularis* ticks from the Calgary Zone (2019). None of the ticks tested were found to harbor *Babesia microti* or *Borrelia miyamotoi*.

Assuming the ticks identified in this sample represent an accurate cross section of species in Alberta, the probability of a tick being removed from a human host (with no travel history) carrying *B. burgdorferi* is estimated to be 0.35% (8/2,326; 95% CI 0.15–0.68%). This value refers to a tick feeding upon a human (at the point of submission before genus and species are known).

## Discussion

This study summarizes overall numbers and the species distribution of ticks found on humans submitted to diagnostic laboratories over a 19-year surveillance period in Alberta, Canada. Analysis of 2,326 tick genera showed, in order of frequency, *Dermacentor* spp.*, Ixodes* spp.*,* and *Amblyomma* spp. This is in stark contrast to findings in Québec and Ontario, where *Ixodes* is the most abundant genus [17,18]. During the surveillance period, the annual proportions of *Ixodes* spp. and *Amblyomma* spp. ticks increased by 2.4% and 2.1%, respectively, and *Dermacentor* spp. decreased by 4.3%.

In comparison to passive surveillance from 2013 to 2019 outlined in the introduction, we received on average less ticks annually (264 ticks per year), of which a lower proportion (3.3%) were found to be able to transmit the agent of Lyme disease. *B. burgdorferi* was detected less frequently on average (4.8%) in the *I. scapularis* or *I. pacificus* ticks tested.

The overall proportion of *B. burgdorferi* positive *I. scapularis* ticks found in our study (12.1%) is lower than most other areas in Canada. A study evaluating *Ixodes* spp. ticks from Ontario and Quebec reported 33–41% positivity for *B. burgdorferi* [19]. In a cross-province Canadian study evaluating ticks from both human and animal sources, the range of *B. burgdorferi* positivity was 9.7–19% from the provinces of Quebec, Manitoba, New Brunswick, Newfoundland, Nova Scotia, Ontario, and Prince Edward Island [20]. Passive and active surveillance of tick populations in Saskatchewan found a rate of 12% [21]. Thus the *B. burgdorferi* positivity found in *I. scapularis* ticks from Alberta is consistent with that seen in other non-endemic areas of Canada, although studies used for this comparison included ticks combined from human, environmental, and veterinary sources. Similar epidemiologic studies evaluating human sources of ticks in Canada have been conducted in the provinces of Manitoba and Ontario. From 2008 to 2012, a surveillance study from Ontario found that 8.4-19.1% of *I. scapularis* submitted were infected with *B. burgdorferi* [17]. Another study evaluating *I. scapularis* from human sources from 2009 to 2016 found that 22.3–25% were infected with *B. burgdorferi* from Manitoba with a lower proportion (12.7–17.6%) positive from Ontario [22]. Both of these provinces are known to be endemic for reproducing *I. scapularis* populations.

The small but significant rise in the number of *Ixodes* spp. in our study (the majority of which are *I. scapularis*) is concerning in light of data supporting a doubling prevalence of tick-borne infections in the last decade [23]. This is likely explained by progressive northward expansion of *I. scapularis* from areas of established populations in the United States, estimated at 46 km/year in a study from Ontario, Canada [24]. Once populations of tick vectors are established, endemicity follows with the rapid emergence of *B. burgdorferi* in these tick populations over a period of several years [25]. This northward expansion is likely multifactorial, including climate change influencing favorable habitat conditions coupled with the increasing availability of hosts, and bird/host migration patterns [26].

Increased numbers of *Ixodes* spp. and *Dermacentor* spp. submissions in our study are not entirely explained by northward expansion, considering the projected timeline [27]. We hypothesize that the occurrence of an extensive wildfire in the areas of Fort McMurray and Wood Buffalo municipalities in northern Alberta between 1 May and 5 July 2016, was an important contributing factor [28]. This fire likely resulted in transient disruptions of local ecosystems leading to shifts in birds/tick host migration within the province with the rise in tick transfer, as well as the movement of tick populations to more metropolitan areas (Edmonton and Calgary Zones) [29–31]. This, along with the increasing involvement of healthcare providers in tick submissions, may explain the higher numbers in these locations. More studies are still required to discern the effect of environmental fires on tick populations [31].

The combination of this data set and ongoing provincial surveillance data (veterinary and environmental submissions) [9], supports the finding that most *Ixodes* spp. ticks are adventitious and reproducing populations of blacklegged ticks (BLTs) do not currently exist in Alberta. This is based on the absence of higher numbers of submissions of BLTs as well as the sporadic finding of *B. burgdorferi* positivity as compared to other endemic locales. Active annual surveillance activities in Alberta from 2014 to 2017 also did not find any *I. scapularis* or *I. pacificus* ticks in the environment [9]. Similar findings are also reported from the neighboring province of Saskatchewan, which partially shares comparable geographic terrain [21].

The lack of *I. scapularis* endemicity in the prairies (outside of Manitoba) supports the low frequency of ticks positive for *Borrelia burgdorferi*, calculated from our data to be 0.35%. This estimate likely represents an over-estimate given data for variables such as tick attachment time and efficiency of pathogen transfer were not available. Furthermore, this numeric is particularly useful for clinicians and public health teams when counseling patients or members of the public experiencing a tick-bite or exposure. While we acknowledge that only *I. scapularis/I. pacificus* were tested for *B. burgdorferi*, it is assumed that the other local species of *Ixodes* ticks would be negative for *B. burgdorferi*. This is a reasonable assumption given major clinical guidelines do not consider other ticks species as significant carriers for this bacterium [32–35]. Such patients generally experience a great deal of anxiety regarding Lyme disease given increased general societal and media awareness [36]. The determination of whether prophylaxis is provided should follow clinical guidelines [32].

A major limitation of this study is that of submission bias – not all ticks on human hosts are routinely submitted, and likely many are discarded after removal. Since 2013, the processes for tick submission to diagnostic laboratories in Alberta have been heavily promoted. Another important limitation is reliance on the provision of travel history on requisitions to determine if a tick was acquired within Alberta. It is not uncommon for requisitions to be incomplete, omit information, or be illegible (ranging from 6% to 32% in several studies) [37-40]. To mitigate this, an aggressive review of archived requisitions was conducted. Despite this, there is a likelihood that some ticks thought to be from within Alberta (based on lack of travel history) are actually imported, such as some listed in Table 1, not normally found in Alberta or Canada (*Ixodes ricinus, Amblyomma cajennense, Amblyomma coelbs*, and *Rhipicephalus pulchellus*). However, these constitute <0.5% of total ticks included. This may also suggest that long-distance travel and exposure to ticks such as *I. ricinus A. cajennense,* etc., is less common than short distance travels to areas where *I. scapularis/I. pacificus* may be endemic. A similar problem may exist for more common ticks that are also found within the province (e.g. *D. variabilis*). Lastly, we do not have complete data available on the number of multiple submissions or numbers of nymphs or larvae. While it is acknowledged these two variables are important in indicating the presence of non-adventitious tick populations, lack of BLT endemicity in Alberta is supported by the low proportion of ticks carrying *B. burgdorferi*, absence of *Ixodes spp.* on provincial active surveillance, and no locally acquired cases of Lyme disease in Alberta [34,41].

The major strength of this study is the long time period covered by the surveillance, allowing analysis of trends over time. While tick identification was carried out at different laboratories, identification and molecular testing of all *Ixodes* spp. was confirmed by a single reference center.

This study represents a retrospective window into a subset of data that is currently not captured in passive tick surveillance in Alberta – the geographic occurrence and prevalence of ticks from human hosts. As such, it provides valuable information to complete data for all hosts for the province. The major genera identified were *Dermacentor, Ixodes*, and *Amblyomma.* The progressive rise in tick submissions over time likely represents increased societal concerns about tick-borne infections (especially Lyme disease), however, the influence of climate change cannot be ruled out. The overall likelihood, of a tick in Alberta carrying the causative agent of Lyme disease is low, which is important to guide patient counseling as well as public health messaging and intervention. Vector species of *Ixodes* ticks will continue to be introduced into the province from neighboring areas. Passive surveillance programs like ours will play an important role in monitoring the occurrence of selected tick species and their implications for public health.

## Author contributions

Jamil N kanji helps in methodology, formal analysis, data curation, writing – original draft, writing – review, and editing. Abraam Isaac helps in formal analysis, validation, resources, writing – original draft, writing – review, and editing. Daniel Gregson helps in investigation, data curation, writing – review, and editing. Monika Mierzejewski helps in formal analysis, data curation, writing – review, and editing. Danny Shpeley helps in investigation, data curation, writing – review, and editing. Pauline Tomlin helps in validation, investigation, data curation, writing – review, and editing. Michael Groeschel helps in investigation, data curation, writing – review, and editing. Robbin Lindsay helps in methodology, investigation, resources, writing – review, and editing. Lisa Lachance helps in writing – review, and editing. Kinga Kowalewska-Grochowska helps in conceptualization, methodology, validation, resources, writing – original draft, writing – review, and editing.

## Supplementary Material

Supplemental Material

## Data Availability

The datasets used and/or analysed during the current study are available from the corresponding author on reasonable request.
