# Supplementary material for: Epidemiology of ticks submitted from human hosts in Alberta, Canada (2000–2019)
Source: Emerg Microbes Infect. 2022 Feb 1;11(1):284–92. doi: 10.1080/22221751.2022.2027217 (PMC8812759; doi:10.1080/22221751.2022.2027217)
Supplement: Supplemental Material [file TEMI_A_2027217_SM4762.docx]

**Supplementary information**

**Manuscript title:**

Low risk of acquiring Lyme disease from a tick bite in Alberta, Canada: surveillance of ticks from human hosts (2000-2019)

**Contents:**

**Supplementary Table S1.** Identification of ticks with associated out-of-province travel history that were not included in tabulation of ticks deemed from within Alberta.

**Supplementary Table S2.** Proportion of each of the major tick genera found based on health zone unit between 2013-2019.

**Supplementary Table S1.** Identification of ticks with associated out-of-province travel history that were not included in tabulation of ticks deemed from within Alberta.

| **Country** | **Province/state**  **(if applicable)** | **Identification**  **(number of ticks)** |
| --- | --- | --- |
| Canada (outside of Alberta) | British Columbia, Manitoba  Northwest Territories, Nova Scotia, Ontario, Saskatchewan | *Dermacentor andersoni* (7)  *D. variabilis* (10)  *Ixodes scapularis* (4) |
| Europe | Czechia, Ireland, Italy, Scandinavia, Scotland (UK) | *D. andersoni* (1)  *I. scapularis* (1)  *I. ricinus* (2)  *Rhipicephalus spp.* (1) |
| United States | Maryland | *I. scapularis* (1) |
| Mexico |  | *D. andersoni* (1) |
| Australia |  | *I. coronatus* (1) |
| Africa | Kenya/Tanzania | *Hyalomma spp.* (1) |
| Central Asia | Khazakstan  Azerbaijan | *I. ricinus* (2) |

**Supplementary Table S2.** Proportion of each of the major tick genera found based on health zone unit between 2013-2019.

| **Tick** | **North Zone** | **Edmonton Zone** | **Central Zone** | **Calgary Zone** | **South Zone** |
| --- | --- | --- | --- | --- | --- |
| *Dermacentor*  (n = 1806) | 4.2 | 11.6 | 11.8 | 65.5 | 6.9 |
| *Ixodes*  (n = 103) | 3.9 | 26.9 | 8.0 | 58.3 | 2.9 |
| *Amblyomma*  (n = 36) | 0 | 27.8 | 16.7 | 55.5 | 0 |
